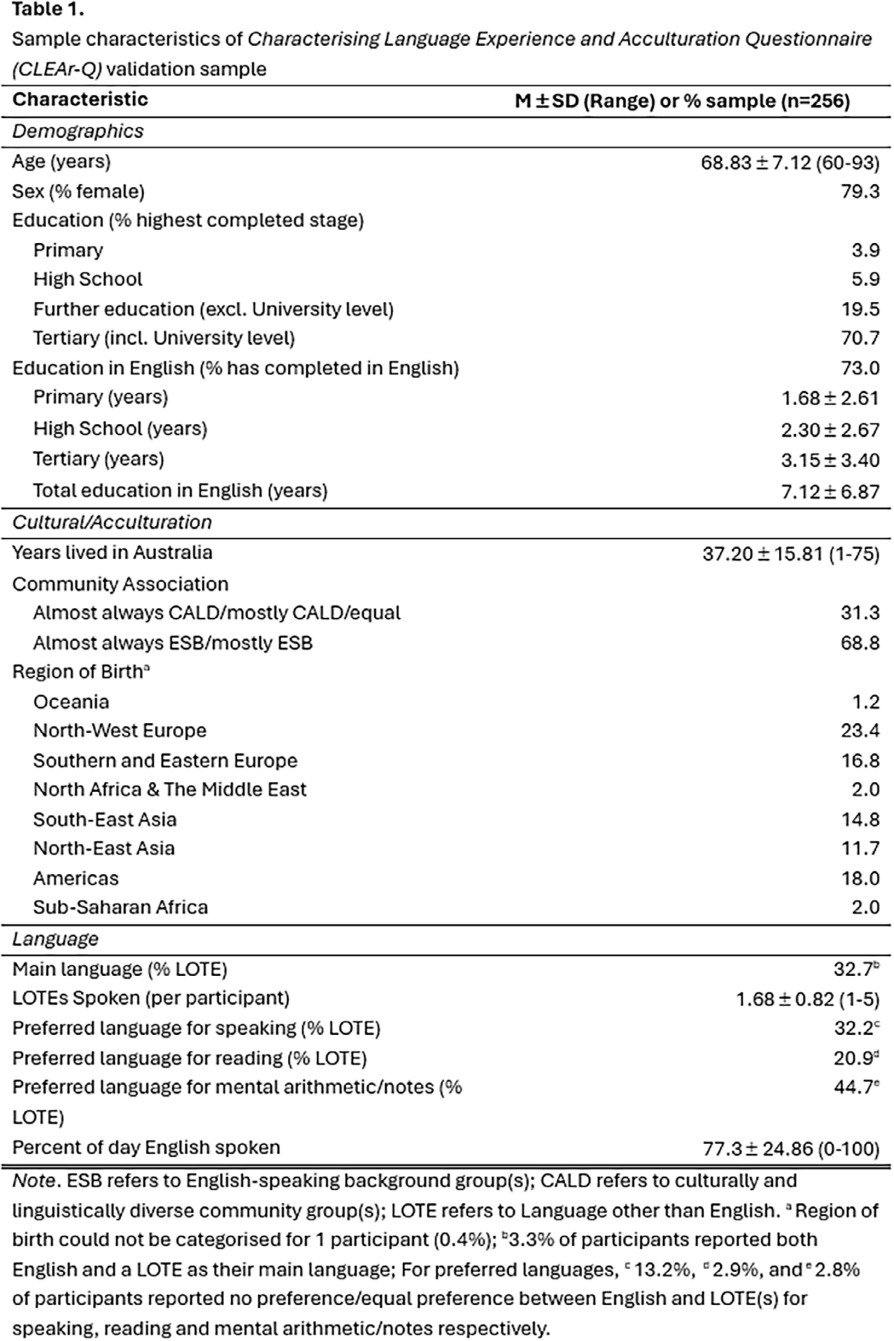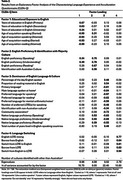# Characterising Language Experience and Acculturation: The factor structure of the *CLEAr‐Q*, a questionnaire to improve the cognitive assessment of culturally and linguistically diverse older adults

**DOI:** 10.1002/alz70857_105358

**Published:** 2025-12-25

**Authors:** Zara A Page, Karen Croot, Ben C.P. Lam, Henry Brodaty, Nicole A. Kochan

**Affiliations:** ^1^ Centre for Healthy Brain Ageing (CHeBA), University of New South Wales, Sydney, NSW, Australia; ^2^ La Trobe University, Melbourne, VIC, Australia; ^3^ Centre for Healthy Brain Ageing (CHeBA), UNSW Sydney, Sydney, NSW, Australia

## Abstract

**Background:**

Linguistic and acculturation variables are known to influence cognitive performance on neuropsychological measures used to assess cognitive impairment and dementia. It is not clear, however, how to best measure these variables in an Australian context to improve cognitive assessment of older adults from culturally and linguistically diverse (CALD) backgrounds. We therefore developed a new measure, the *Characterising Language Experience and Acculturation Questionnaire (CLEAr‐Q)* and used Exploratory Factor Analysis (EFA) to determine the underlying factor structure.

**Method:**

An online version of the *CLEAr‐Q* was developed using items drawn or modified from the literature with consultation from a CALD community working group via an adapted participatory research framework. It was administered in English via an anonymous survey. From the initial 52 items, 31 were considered for further analysis. EFA with oblique rotation (geomin) was used.

**Result:**

The validation sample included 256 participants aged 60‐93 (Table 1) born outside of Australia and reporting a native Language Other Than English (LOTE). EFA suggested a four‐factor solution: Educational Exposure to English, English Proficiency and Identification with Majority Culture, Dominance of English Language and Culture, and Language Switching. The model fitted well with the data, with Comparative Fit Index (CFI) = 0.98 and Standardised Root Mean Square Residual (SRMR) = 0.07. In combination, these factors explained 58.9% of the variance. Table 2 presents factor loadings.

**Conclusion:**

The present study demonstrates the utility of the *CLEAr‐*Q in characterising the diversity in linguistic and acculturation variables in an Australian CALD sample. Our results also inform item selection for a streamlined version of the *CLEAr‐Q*. The factor structure provides evidence for the interwoven relationship between linguistic and acculturation variables and supports the need for a comprehensive tool, rather than simple proxies, to understand the influence of cultural and linguistic diversity on cognitive assessment. In future work we will investigate the relative importance of the four *CLEAr‐Q* factors in predicting cognitive performance, which is expected to improve diagnostic accuracy of current neuropsychological measures in assessing cognitive impairment and dementia in older adults from CALD backgrounds.